# Structure, Function, Pharmacology, and Therapeutic Potential of the G Protein, Gα_/q,11_

**DOI:** 10.3389/fcvm.2015.00014

**Published:** 2015-03-24

**Authors:** Danielle Kamato, Lyna Thach, Rebekah Bernard, Vincent Chan, Wenhua Zheng, Harveen Kaur, Margaret Brimble, Narin Osman, Peter J. Little

**Affiliations:** ^1^Discipline of Pharmacy, Diabetes Complications Group, School of Medical Sciences, Health Innovations Research Institute, RMIT University, Bundoora, VIC, Australia; ^2^State Key Laboratory of Ophthalmology, Zhongshan Ophthalmic Centre, Guangzhou, China; ^3^Faculty of Health Sciences, University of Macau, Macau, China; ^4^Department of Chemistry, University of Auckland, Auckland, New Zealand

**Keywords:** G proteins, GPCR, cell signaling, therapeutic targets, transactivation

## Abstract

G protein coupled receptors (GPCRs) are one of the major classes of cell surface receptors and are associated with a group of G proteins consisting of three subunits termed alpha, beta, and gamma. G proteins are classified into four families according to their α subunit; Gα_i_, Gα_s_, Gα_12/13_, and Gα_q_. There are several downstream pathways of Gα_q_ of which the best known is upon activation via guanosine triphosphate (GTP), Gα_q_ activates phospholipase Cβ, hydrolyzing phosphatidylinositol 4,5-biphosphate into diacylglycerol and inositol triphosphate and activating protein kinase C and increasing calcium efflux from the endoplasmic reticulum. Although G proteins, in particular, the Gα_q/11_ are central elements in GPCR signaling, their actual roles have not yet been thoroughly investigated. The lack of research of the role on Gα_q/11_ in cell biology is partially due to the obscure nature of the available pharmacological agents. YM-254890 is the most useful Gα_q_-selective inhibitor with antiplatelet, antithrombotic, and thrombolytic effects. YM-254890 inhibits Gα_q_ signaling pathways by preventing the exchange of guanosine diphosphate for GTP. UBO-QIC is a structurally similar compound to YM-254890, which can inhibit platelet aggregation and cause vasorelaxation in rats. Many agents are available for the study of signaling downstream of Gα_q/11_. The role of G proteins could potentially represent a novel therapeutic target. This review will explore the range of pharmacological and molecular tools available for the study of the role of Gα_q/11_ in GPCR signaling.

## Introduction

G protein coupled receptors (GPCRs) constitute the largest class of cell surface receptors. GPCR genes account for 5% of the human genome (1, 2). Of these receptors, all are seven membrane spanning receptors but not all are G protein binding but it is convenient to refer to the receptors as GPCRs. GPCRs also represent the largest and among the most efficacious class of therapeutic targets for diseases including cardiovascular disease, cancer, and asthma (1, 2). Many drugs have been developed based on GPCRs and these include some of the most important agents in human medicine, for example, in the treatment of asthma and hypertension (3). GPCRs are helical transmembrane receptors complemented by functional extracellular and intracellular loops (4). Within the GPCR superfamily, there have been five major families identified. They are the rhodopsin, secretin, glutamate, adhesion and frizzled/taste2 families (5). Most GPCRs contain seven helices and three intracellular loops; however, some members of the rhodopsin family may have eight helices and four intracellular loops (6). GPCRs bind hormones, neurotransmitters, or growth factors (7), which initiate a plethora of cellular responses. GPCRs are generally ligand activated but they can also bind to Gα-subunits in the absence of a ligand, a phenomenon known as receptor pre-coupling. GPCRs interact with their respective G proteins only upon receptor activation known as the collision coupling model or in the absence of agonist known as the pre-coupled receptor model (8).

Whereas protein tyrosine and serine/threonine kinase receptors have intrinsic catalytic activity, GPCRs do not have enzymatic activity *per se* but are linked to Gα proteins, which are GTPases, and mediate the signal transduction (9). G proteins of the α, β, and γ families provide the specificity and functionality of GPCRs.

**Figure 1:**
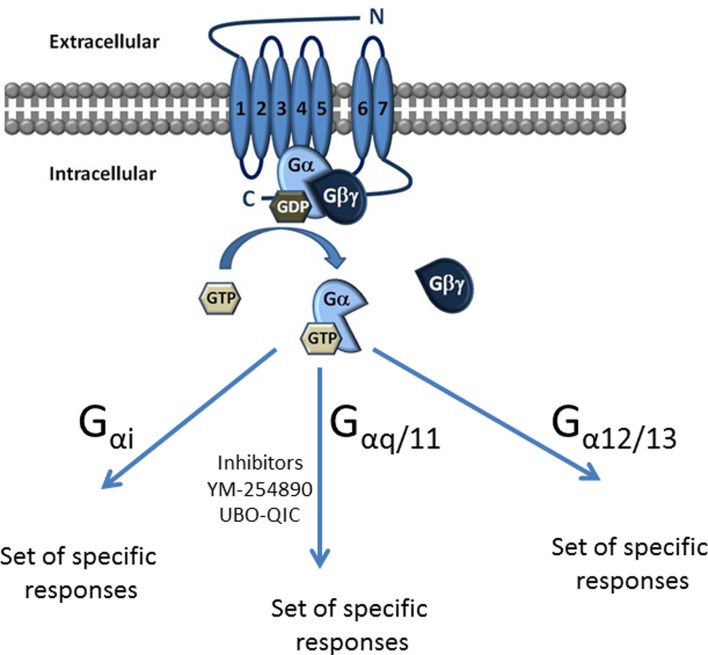
**Classification of G proteins into four families according to their α subunit**. The Gα subunit is made up of Gαs, Gαi, Gαq/11, and Gα12/13. Gαs and Gαi families regulate adenylyl cyclase activity, while Gαq activates PLC-β and the Gα12/13 can activate small GTPase families. The Gαq subunit is made up of four members, which include the Gαq, Gαq/11, Gαq/14, and Gαq15/16.

G proteins are classified into four families according to their α subunit: G_i_, G_s_, G_12/13_, and G_q_ (Figure 1). The G_s_ and G_i_ families regulate adenylyl cyclase activity, while G_q_ activates phospholipase Cβ and G_12/13_ can activate small GTPase families (10). The G_q_ family consists of four members: G_q_, G_11_, G_14_, and G_15/16_ (11, 12) and their respective α subunits are thus Gα_q_, Gα_11_, Gα_14_, and Gα_15/16_ (Figure 1).

The role of G proteins in GPCR signaling has not been as intensively investigated as other aspects of GPCR signaling possibly due to the limited availability of convenient pharmacological tools. The most useful pharmacological agent has been the compound known as YM-254890, which is a cyclic depsipeptide isolated from the *Chromobacterium* sp. Initial studies indicated that this is a specific inhibitor of Gα_q/11_. YM-254890 has had variable availability and has not been available in recent times. As the importance of GPCR signaling in physiology and pathophysiology continues to grow, the potential importance of G proteins increases both for the fundamental cell biology and as potential therapeutic targets.

One of the major and expanding areas of GPCR signaling is transactivation-dependent signaling (13) in which GPCRs transactivate protein tyrosine kinase (PTK) and protein serine/threonine kinase receptors (14–16). Transactivation greatly expands the roles of GPCRs in cell biology (13, 17–19). GPCR transactivation of PTK receptors was discovered in 1996, has been the subject of almost 200 publications, and has been recently reviewed (20). Our laboratory has recently extended the paradigm of GPCR to PTK receptor transactivation to include the transactivation of protein serine/threonine kinase receptors and specifically the protease-activated receptor (PAR)-1 and endothelin receptor (ETR)-mediated transactivation of the transforming growth factor (TGF)-β type I receptor (TGFBR1) also known as Activin-like Kinase (Alk)-V (15, 16, 21). There is very little information on the role of Gα proteins in GPCR transactivation signaling. There is a need for synthetic programs to provide new molecules with the pharmacological properties of YM-254890 and such programs will provide agents, which allow for a much broader range of studies on the role of G proteins in GPCR signaling. This review focuses on the role of Gα_q/11_ in GPCR signaling in the context that the availability of new tools will shortly lead to a large increase in studies in this area. The two targets of compound such as YM-254890 are Gα_q_ and Gα_11_ – these two proteins are distinct gene products but they have an identical number of amino acids and essentially identical structures and functions. In this review, we refer to Gα_q_ but most statements will also relate to Gα_11_ and only where differences are known and of significance will this distinction be drawn.

## Gα_q/11_ Signaling

The responses to GPCR agonists and the conformational changes in the GPCR that are induced by ligand binding are transduced and then mediated by heterotrimeric G protein complexes. Consisting of three subunits α, β, and γ, their role is to transduce external stimuli into intracellular signaling cascades. Most of the specificity of signaling resides in the Gα subunit. In an inactivated state, the α subunit binds guanosine diphosphate (GDP); however, upon binding activation of the GPCR, GTPase activity is induced and promotes the exchange of bound GDP for guanosine triphosphate (GTP). The α subunit and βγ complex then dissociate from one another and interact with their associated effectors (22). In the most common signaling pathways, Gα_q_ activates phospholipase Cβ (PLCβ), which hydrolyzes phosphatidylinositol 4,5-bisphosphate (PIP_2_) releasing diacylglycerol (DAG) and 1,4,5-inositol trisphosphate (IP_3_). DAG activates a number of isoforms of protein kinase C (PKC), whereas IP_3_ diffuses to the endoplasmic reticulum (ER) and binds to IP_3_ receptors on ligand-gated calcium channels on the surface of the ER leading to a massive release of calcium ions into the cytosol and subsequently in some cells, the opening of cell surface calcium channels leading to the influx of extracellular calcium (23). The calcium cycle continues with the uptake of calcium back into the ER by Ca ATPases.

In addition to this paradigm, it has been shown that RhoA is a mediator of calcium sensitization and is downstream of Gα signaling. Activation of the members of the Rho family is via GTP binding. The exchange of GDP for GTP on these proteins is controlled through guanine nucleotide exchange factors (GEFs), which catalyze the exchange of GDP for GTP (24). Activation of Rho-mediated signaling pathways can be indirectly mediated by GPCRs, integrins, or receptor tyrosine kinases. G proteins, Gα_12_ and Gα_13_, activate Rho by activation of a Rho GEF (25). It is only when RhoA is active that it can interact with and activate downstream effectors such as Rho kinase (ROCK).

Thrombin activation of PAR-1 involves both Gα_q/11_ and Gα_12/13_, which causes RhoA activation signaling downstream to stimulate ROCK and PKC-related kinase. RhoA activation coupled to Gα_q/11_ involves intracellular release of calcium involving the downstream activation of the two Rho-regulated protein kinases, which in turn regulates the contraction of actomysin and the formation of focal adherence in human endothelial cells. In the case of Gα_12/13_, RhoA is activated through GEFs such as p115 RhoGEF, PDZ-RhoGEF, or leukemia-associated RhoGEF. In the case of Gα_q/11_, it is suggested that the GEFs utilized may involve p63 Rho GEF or Trio; however, the specific GEFs involved in this signaling pathway are yet to be confirmed.

Overexpression of active Gα_11_ or stimulation of the m1 muscarinic acetylcholine receptor induces apoptosis in HeLa cells. Rho kinase and ROCK are stimulated due to the cleavage of activated caspase 3 during apoptosis. There have been several studies on the mechanisms involved in Gα_q/11_-induced apoptosis, which show that this phenomenon is cell- and context-dependent. In COS-7 and CHO cells, Gα_q_-induced apoptosis is dependent on PKC, and angiotensin II-induced myocyte apoptosis is dependent on the release of intracellular calcium suggesting the involvement of PLC pathway. The molecular mechanism of Gα_q/11_ induced apoptosis leading to the activation of Rho/ROCK is not clearly understood; however, some studies have shown that Gα_q/11_ signaling activated RhoA, which inhibited insulin-stimulated Akt phosphorylation in HeLa cells. In CHO cells, Gα_q_ and Gα_11_ regulate actin cytoskeleton remodeling through the activation of ADP-ribosylation factor 6. Platelets stimulated with P2Y1 agonist leads to the activation of RhoA, this activation was inhibited by Gα_q_ inhibitor YM-254890, indicating that RhoA activation downstream of purinergic (P2Y)-1 receptors requires Gα_q_ stimulation (26).

## Structure of Gα_q_

Gα_q_ and Gα_11_ are distinct gene products but from the same chromosome (12). These two proteins have an identical number of amino acids and are functionally almost identical. However, the tissue distribution of the two isoforms is distinct (12). Gα_q_ is a 359 amino acid protein comprising two domains: a helical domain and a GTPase binding domain. The GTPase domain is responsible for hydrolyzing GTP to GDP, as well as binding the Gβγ subunits, GPCRs, and other effectors. This domain is conserved between all members of the G protein superfamily (6). The GTPase domain contains three switch regions, which are flexible loops that change conformation when bound with GTP. The helical domain contains six α-helices, which encapsulates nucleotides in the protein core by forming a lid over the nucleotide-binding pocket. Of all G protein families identified, members of the Gα_q_ family share the most amino acid sequence homology. In humans, Gα_11_, Gα_14_, and Gα_16_ share 90, 80, and 57% sequence similarities, respectively (27).

## Functions of Gα_q_

Gα_q_ plays a role in platelet aggregation. Bleeding time and resistance to thromboembolism are dramatically increased in Gα_q_-deficient mice compared to wild type (28). Gα_q_ is also implicated in insulin-stimulated glucose transport (29). In 3T3–L1 adipocytes, Gα_q_ is required for insulin-induced GLUT4 translocation and the stimulation of 2-deoxy-d-glucose uptake. Angiotensin II dose-dependently increases cell proliferation in smooth muscle cells and this is inhibited by the Gα_q_ antagonist, GP-2A (30). Gα_q/11_ proteins are involved in HIV-1 envelope glycoprotein-dependent cell–cell fusion upstream of Rac-1 (31). Genetically modified mice studies suggest that receptors coupled to the Gα_q_ play a role in the development of heart failure (32). Following treatment to activate Gα_q_ in transgenic mice expressing a silent Gα_q_, the mice rapidly developed a dilated cardiomyopathy and heart failure. Transgenic mice expressing an inducible Gα_q_ that cannot activate PLCβ do not develop heart failure. Thus, the activation of Gα_q_ resulting in heart failure requires the activation of PLCβ (32).

## Role of Gα_q_ in the GPCR Transactivation of Kinase Receptors

There are now two major pathways of GPCR to cell surface receptor kinase transactivation – the well-established transactivation of PTK receptors, notably epidermal growth factor receptor (EGFR) and the recently identified transactivation of serine/threonine kinase receptors, specifically the TGFBR1 (14–16, 33). There is some information of the role of Gα proteins and thus Gα_q_ in the transactivation of PTK receptors but nothing is known of the role of Gα_q_ in the transactivation of serine/threonine kinase receptors.

G protein coupled receptors coupled to Gα_q_, such as bombesin receptor or Gα_i_ proteins, such as M2 muscarinic acetylcholine receptor, expressed in COS-7 cells show increased EGFR tyrosine phosphorylation more than that resulting from Gα_i_ coupled receptor stimulation. Cells transfected with Gα_q_-coupled GPCRs are unaffected by pertussis toxin while Gα_i_ coupled receptors are, as expected, blocked by pertussis toxin treatment (34). Thus, EGFR transactivation may occur through both pertussis toxin-sensitive and -insensitive pathways. GPCR transactivation of serine/threonine kinase receptors and specifically TGFBR1 by both ETR and PAR-1 has been identified in vascular smooth muscle cells (VSMCs) but the role of Gα_q_ in transactivation of TGFBR1 has not been reported (15, 16, 21).

Thus far, the biochemical mechanisms of GPCR to protein tyrosine and protein serine/threonine kinase receptors have been found to be completely distinct with, for example, the former involving MMPs and the latter being independent of MMPs (16). The transactivation of serine/threonine kinase but not tyrosine kinases involves the cytoskeleton (16). The independent signaling pathways have made it difficult to envisage a single potential therapeutic target for the inhibition of all GPCR transactivation signaling (18).

It will be interesting to investigate the role of Gα_q_ proteins in tyrosine and serine/threonine kinase transactivation signaling as it has the potential to be a point of commonality in GPCR-mediated transactivation of cell surface protein tyrosine and serine/threonine kinase receptor signaling.

## Molecular and Pharmacological Regulation of G Proteins

G proteins in cells can be effectively knocked down utilizing a molecular approach and this has allowed for detailed studies of the function of various G proteins and their interactions. Classic experimental approaches assume that the intervention is specific and does not alter other parameters that would impact on the experimental result of the index intervention. This is not always the situation and is certainly not the reliable paradigm in the case of the regulation of G proteins. Gilman and colleagues (35) demonstrated that knocking down Gβ proteins resulted in a compensatory increase in both the effector, adenylyl cyclase and even the GPCR, being the β2-adrenergic receptor. Results of knock down interventions are also not always reciprocal – the knock down of one G protein may lead to a compensatory increase in another G protein family member but the reverse or reciprocal phenomenon may not occur (35). Thus, the knock down of Gα_q_ and Gα_11_ in HeLa cells increased the accumulation of Gα_i_ and Gα_0_ but the reciprocal response did not occur (35).

Gα and β proteins exist in approximately equal mass stoichiometry in most cells. This occurs primarily because Gβ proteins stabilize bound Gα proteins with the corollary that free Gα proteins are degraded. However, Gα proteins are subject to palmitylation and myristoylation and these processes may bind Gα proteins to the cell membrane and stabilize the proteins (36). A consequence of the role of post-translational regulation on stability and the cellular levels of G proteins is that the relationship between mRNA and protein levels may be perturbed. Higher mRNA levels may lead to increased expression of the G protein, but if it is orphaned and free the protein may be degraded providing for high level of mRNA and in the presence of low levels of protein.

Molecular approaches to the up- and down-regulation of target proteins are a major component of modern mechanistic studies of cell biology. However, as exemplified above, alteration of target protein levels may result in compensatory changes in other components of a system and the perturbation might not provide the expected result. Pharmacological approaches nullify the activity or function of a target protein without in most cases altering the level of the target protein. If there is greater availability of G protein inhibitors such as YM-254890 or alternative new tools, then it will be interesting to determine if blocking a Gα protein results in any changes in the level of other G proteins within the cell. Such studies are currently underway in our laboratory.

## Pharmacology of Gα_q_ Inhibitors

### YM-254890

The compound known as YM-254890, a cyclic depsipeptide isolated from the *Chromobacterium* sp. QS3666, is a specific Gα_q_ inhibitor. YM-254890 has been shown to inhibit ADP-induced platelet aggregation, which is mediated via GPCRs, P2Y_1_, and P2Y_12_ (37). These receptors are associated with the Gα_q_ and Gα_i_ signaling pathways, respectively. YM-254890 has no effect on the P2Y_12_ signal transduction pathway, indicating that the compound has some specificity for Gα_q_. It was also shown to inhibit Gα_q_-coupled GPCR signaling by inhibiting calcium mobilization in P2Y_2_-expressing C6-15 cells but not cAMP accumulation (38).

YM-254890 inhibits the signal transduction of Gα_q_ by inhibiting the exchange of GDP for GTP preventing the activation of the G protein, rather than receptor-Gα_q_ interactions (38). When bound to GDP, the non-polar side chains of YM-254890 form hydrogen bonds with the Switch I region; however, this is a conformation that cannot be maintained when bound with GTP (39). Aside from antiplatelet activity, by electrically inducing carotid artery thrombosis in rodents, YM-254890 was also shown to have antithrombotic and thrombolytic effects (40).

YM-254890 was discovered and developed by Yamaguichi Pharmaceuticals, Japan; Yamaguichi subsequently became the property of Astellas Pharmaceuticals, Japan. YM-254890 was made available to researchers 10 years ago and a small number of interesting studies were published. The initial results indicated that YM-254890 is a useful tool for investigating Gα_q/11_-coupled receptor signaling and the physiological roles of Gα_q/11_. For example, Gα_q_ knockout mice have lower blood pressure than appropriate controls (41). This indicates some potential for a Gα_q_ inhibitor to be an anti-hypertensive agent and accordingly YM-254890 has not been provided to researchers presumably because of such identified commercial value. As discussed above, molecular approaches in this area, for example, G protein knock down can lead to rebound increases in other G proteins with unexpected results. Accordingly, it is understood that a number of groups are undertaking programs for the synthesis of compounds related to YM-254890 and it is likely that the availability of potent and specific Gα_q/11_ inhibitors would greatly expand activity and knowledge in this area and answer important questions such as the role of Gα_q/11_ in GPCR transactivation signaling of protein kinase receptors.

### UBO-QIC/FR300359

FR300359, henceforth referred to as UBO–QIC, is also, like YM-254890 a cyclic depsipeptide; it is isolated from the *Ardisia crenata sims* plant (42). UBO-QIC is structurally very similar to YM-254890 and not surprisingly shows similar pharmacological activity. UBO-QIC inhibits platelet aggregation in rabbits *in vitro* and causes dose-related hypotension in anesthetized normotensive rodents, which is consistent with the effect on blood pressure in Gα_q_ knock down mice (41, 43). The blood pressure lowering effect was attributed to the ability of UBO-QIC to partially mediate nitric oxide release from endothelial cells and inhibit calcium migration caused by voltage-dependent and receptor-operated channels (44).

Since the discovery of UBO-QIC as a Gα_q_ antagonist, there have been limited studies showing its use. In HEK cells transfected with TRPV4, PAR-2-mediated intracellular calcium release was abolished by UBO-QIC when compared to control; however, extracellular calcium influx through the TRPV4 ion channel was unaffected thus showing that PAR-2 coupling to TRPV4 is not mediated by Gα_q_ signaling (45). There have been no studies directly comparing the activity of YM-254890 and UBO-QIC possibly because of the linked variability of the former compound whereas at the time of preparing this review, UBO–QIC is commercially available.

## The Peptide Antagonist GP-2A

In 2004, Tanski et al. (30) discovered a competitive Gα_q_ inhibitor, G Protein antagonist-2A, also known as GP-2A. GP-2A is a peptide that selectively inhibits the action of Gα_q_ by M1 muscarinic cholinergic receptors. The signaling pathway of Gαq and its role in cell proliferation with rat pulmonary artery smooth muscle cells were studied. Angiotensin II-mediated proliferation, PLCβ activation, and Erk1/2 phosphorylation were inhibited by more than 50% in the presence of GP-2A (30). The EGFR can be activated by EGF to generate an intracellular signaling pathway leading to the phosphorylation of several downstream effector proteins such as Erk1/2 (46). Tanski and colleagues have evaluated angiotensin II (as specific Gα_q_ agonist) to effectively reduce Erk1/2 activation mediated by PLCβ via Gα_q_ in the presence of GP-2A by showing its association with the phosphorylation of Erk1/2 in rat pulmonary artery smooth muscle cells (30). This study provides a strong foundation for our laboratory research as we can further investigate the possibility of this downstream signaling pathway to see whether or not GP-2A can act on other GPCR agonists such as thrombin to effectively respond similarly via Gα_q_ in other smooth muscle cell types such as human VSMCs.

## Other Pharmacological Tools for Evaluating the Role of Gα_q_ in GPCR Signaling

**Figure 2:**
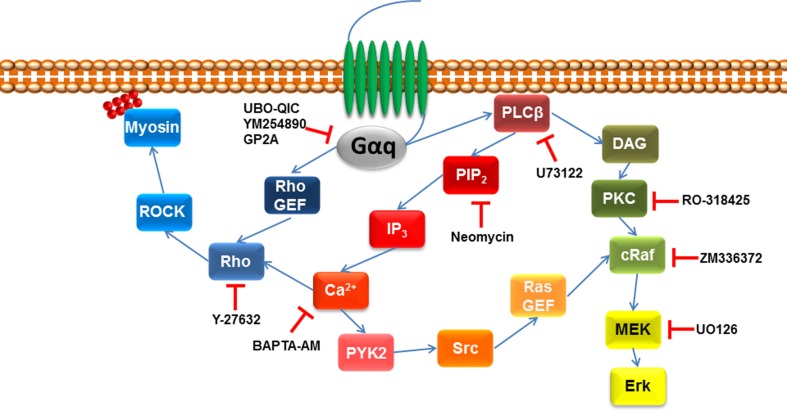
**Pharmacological agents to inhibit downstream signaling intermediated of Gαq**. Once GPCR is activated by its agonist, Gαq signaling activates phospholipase C β (PLCβ), which leads to the hydrolysis of phosphatidylinositol 4,5-biphosphate (PIP_2_) and diacylglycerol (DAG). The former leads to initiate the release of 1,4,5-inositol tris phosphate (IP_3_) initiating calcium release, activating protein tyrosine kinase 2 (PYK2), which leads to proto-oncogene tyrosine protein kinase (Src) activating Ras guanine nucleotide exchange factor (Ras GEF), which leads to the activation of MAPK signaling. MAPK signaling pathway can also be downstream of DAG that activates protein kinase C (PKC), which leads to the activation of MAPK signaling. Gαq signaling can also go indirect of PLCβ by activating Rho GEF leading to the activation of the Rho/ROCK signaling pathway.

It is also possible to indirectly assess the role of Gα_q_ in GPCR signaling by analyzing downstream events through the use of inhibitors (Figure 2). For example, as detailed above GPCR ligand engagement activates Gα_q_ which in turn activates phospholipase C leading to the catalysis of PIP_2_ and the release of DAG and IP_3_. There are inhibitors of PLC–β including U73122, and its inactive analog U-73343 is available to use as a control compound. These compounds have been widely used (47–51) although they are not considered to be especially useful and specific agents. The antibiotic, neomycin, can also be used as a PLCβ inhibitor in that it binds to the target substrate, PIP_2_ and inhibits the action of PLCβ to release DAG and IP_3_, which can be assessed as a reduction in IP_3_ accumulation or increased free intracellular calcium (23). As always with pharmacological approaches, it is likely that the use of multiple approaches can provide the best information on the role of Gα_q_ in GPCR signaling (Table 1).

**Table 1:** **Pharmacological tools used as inhibitors of Gαq or its downstream signaling intermediates**.

	Inhibitors	Actions	Reference
Direct	YM-254890 UBO-QIC	Gαq inhibitor by inhibiting the exchange of GDP for GTP preventing the activation of the G protein	(37) (42)
	GP-2A	Competitive Gαq inhibitor	(30)
Downstream	U73122/U73343	PLC-β inhibitors	(47)
	Neomycin	Binds to PIP_2_ and blocks the action of PLC-β	(23)
	BAPTA-AM	A chelator of calcium ions reventing increase in intracellular calcium	(52)
	Y-27632	RhoA/ROCK specific antagonist	(53)

## U73122 and Its Inactive Analog U-73343

To maximize our knowledge of Gα_q_, it is possible to examine the downstream role of Gα_q_ in GPCR by assessing the inhibitors of PLC, U73122, and its inactive analog U73343. U73122 and its analog U73343 were used to show the effect of human platelet calcium signaling and protein tyrosine phosphorylation in the presence of thrombin, collagen, and thapsigargin (47). U73122 showed complete inhibition of calcium signaling in the presence of this agonist, which was generated via the activation of PLC specifically the β and γ isoforms (47, 54). U73343 did not show any calcium inhibitory effect via the activation of PLC but rather showed the calcium inhibitory effect via the upstream activation of cPLA2 in the presence of thapsigargin and collagen (47). This provides a clear indication that U73343 has minimal activity as a PLC inhibitor.

The study also investigated the role of Gα_q_ in the transactivation of PTK receptors to show that platelets, stimulated by thrombin increased protein tyrosine phosphorylation. In the presence of U73122, the phosphorylation of tyrosine kinase was abolished (47). As mentioned earlier, Gα_q_ are prominent in signaling in VSMCs. From the recent study, it is possible that we can replicate this investigation in other cell type to further study the PTK and perhaps the serine/threonine kinase transactivation pathway in the presence of U73122.

## Neomycin and Its Potential Role for Gα_q_ Signaling Studies

As mentioned above, another agent, which can further be investigated, is neomycin, a PLC inhibitor. Our laboratory has previously reported that neomycin strongly inhibits the formation of IP_3_ in rat aortic smooth muscle cells in the presence of endothelin, an agonist that influences contraction in smooth muscle (23). Endothelin acts via specific ET-A receptors, which are coupled to PLC to stimulate calcium mobilization (23). ETR is coupled to PLC via G proteins (55) and its activation acts on the cardiac muscle where it binds to ryanodine located on the SR, which releases calcium mobilization within the cardiac muscle cell (56). As known, the signaling pathways of GPCRs through G proteins contribute to various functions in different cell types such as the contraction of blood vessels and are involved in many diseases such as cancer and cardiovascular disease (16, 57). In unpublished data, we have found that neomycin has a dose-dependent inhibition of thrombin-mediated release of intracellular calcium in human VSMCs. Further investigation is required to understand its pharmacological signaling pathway via Gα_q_.

## The Action of Intracellular Calcium Ion to Study the Role of Gα_q_ in GPCR Signaling

The activities of calcium ions on the inhibitory action of calcium channel blockers and its impact on atherogenesis in the regulation of proteoglycan biosynthesis in human VSMCs was studied via the role of ionomycin and bis (2-amionophenyl) ethyleneglycol-*N,N,N*′,*N*′-tetraacetic acid, tetraacetoxymethyl ester (BAPTA-AM) (58).

Ionomycin is a calcium ionophore, which elevates intracellular calcium (58). Radioactive sulfate incorporation into proteoglycans was unaffected by ionomycin, providing support that calcium regulation is not involved in proteoglycan synthesis (58).

Similarly, BAPTA-AM, a chelator of calcium ions, which prevents an elevation of intracellular calcium by acting as a calcium buffer (52) had no effect on proteoglycan synthesis (58). Agonists, TGF-β, and ET-1 stimulated BAPTA-AM to decrease sulfate incorporation into proteoglycans. The interpretation therefore concluded that there were no effects on calcium ion stimulation hence intracellular calcium does not play a role in VSMC proteoglycan synthesis (58).

## Y-27632 – A RhoA/ROCK Inhibitor

Y-27632 is a widely used specific inhibitor of RhoA/ROCK family of protein kinases (53). The ROCK family of kinases is involved in Rho-induced formation of actin-stress fibers and focal adhesion as well as the down-regulation of myosin light chain (MLC) phosphatases. Deng et al. (59) examined the role of the PLC calcium pathway and Rho Kinase in PAR-1-mediated CCL2 release. Rho kinase activation is mediated by a Gα_q_-PLC-calcium-dependent PKC pathway to release thrombin-mediated CCL2. Thrombin-induced phosphorylation of MLC was inhibited by PLC calcium and calcium-dependent PKC inhibitors. Q94 a PAR-1 selective Gα_q_ antagonist abolished thrombin-mediated MLC phosphorylation. Subsequent experiments showed that blockade of Rho kinase signaling is not essential for CCL2 protein production but is important in the release of CCL2 from the cell, as thrombin-mediated CCL2 levels are inhibited by Y-27632.

Having provided evidence that cytoskeletal rearrangement is involved in thrombin-mediated transactivation of TGFBR1, ROCK inhibitor Y-27632 was used to study the role of ROCK in the transactivation signaling pathway. Y-27632 inhibited the downstream product of ROCK-phosphor-Ezrin, Radixin, and Moesin. The ROCK inhibitor abolished the thrombin-mediated increase in phosphoSmad2, indicating that Y-27632 inhibits the activity of ROCK and ROCK is involved in the thrombin-mediated transactivation of TGFBR1. To evaluate the role of ROCK signaling in cardiac contractility, hearts were treated with Y-27632. This lead to a significant inhibition in the peak of pressure of non-transfected hearts but no reduction in basal contractility in hearts overexpressed with α1A-adrenergic receptor signifying that ROCK pathways play an important physiological role in maintaining baseline contractility (60).

## Gα Gene Knockdown Using siRNA

Despite the very large number of GPCRs, there are relatively few studies that have used the potential of Gα_q/11_ gene knockdown by siRNA to explore their roles in the signaling cascades. One of the first reported gene knockdown studies of Gα proteins was the knockdown of Gα_q_ and Gα_11_ gene expression using siRNA in HeLa cells (35). This work demonstrated an absolute requirement of Gα_q/11_ to stimulate histamine-mediated phospholipase C activity. Silencing of Gα_q_ or Gα_11_ caused indistinguishable phenotypes, loss of half of histamine-stimulated PLC activity, despite the fact that concentrations of Gα_11_ exceed those of Gα_q_ by 10-fold. No compensatory increases of either Gα_q_ or Gα_11_ were observed following loss of either protein. Loss of Gα_q_ or Gα_11_ did cause increased accumulation of Gα_i_ and Gα_o_ (35). A study characterizing the Gα subunits required for PAR-1-mediated endothelial cell permeability showed that both Gα_q_ and Gα_11_ were necessary for thrombin to increase permeability while the need for Gα_12/13_ was less. Both protein subunit families contributed significantly to RhoA activation by thrombin (61). Knockdown of Gα_q/11_ in human pulmonary artery smooth muscle cells alters but does not prevent hypoxia-induced mitogenic factor-mediated calcium release demonstrating that Gα_q/11_ contributes to hypoxia-induced PLC signaling pathway (62). Using siRNA knockdown of Gα_q_ or Gα_s_ in human prostate epithelial cells, GPCR melatonin receptor MTNR1A has a dual requirement of Gα_q_ and Gα_s_ receptor coupling for effective MTNR1A signal transduction (63). In HEK cells expressing high levels of thyrotropin-releasing hormone receptor 2, knockdown of Gα_q/11_ reduces persistent agonist-induced signaling by 82% and suggests that Gα_q/11_ is a required component of the activated receptor signaling pathway (64). Clearly, there is considerable scope to use siRNA technology more often as a very useful tool in delineating the importance of Gα proteins in GPCR signaling.

## Gα Knockout Mice and Gα Protein Mutants and Chimeras

Other molecular approaches to investigate the varied roles of Gα proteins include generating Gα protein knockout mice or overexpressing a variety of Gα mutants and chimeras in cell lines and examining their effects in different cellular contexts.

Over the last decade, the most widely used Gα knockout model is the Gα_q/11_ knockout mouse. Gα_q/11_-deficient fibroblasts from these knockout mice have been used to study a large number of GPCR signaling pathways. These studies have demonstrated that Gα_q/11_-deficient mouse fibroblasts expressing bradykinin B (2) receptor require both Gαi and Gα_q/11_ for effective bradykinin-mediated stimulation of the Erk cascade (65). Gα_q/11_ knockout mouse fibroblasts expressing GPCR α1b-adrenoreceptor protein or fusion proteins consisting of the α1b-adrenoreceptor and wild-type Gα_q/11_ or palmitolyation-resistant Gα_q/11_ mutants reveal agonist-mediated receptor/Gα_q/11_-coordinated release of the βγ complex (66). Expressing fusion proteins consisting of the GPCR α1b-adrenoreceptor with various Gα mutants results in altered receptor contact domain residues and enables the identification of key agonist and antagonist receptor contact sites that are necessary for α1b-adrenoreceptor activation. An aromatic group four amino acids before the carboxy terminus in Gα_q/11_ provides maximal α1b-adrenoreceptor activation information (67). Mouse embryonic fibroblasts from double knockout Gα_q/11_ and β-arrestin mice demonstrated that kisspeptin activation of GPCR 54 regulates the hypothalamic–pituitary–gonadal axis in reproductive function and that GPCR 54 has a co-dependency of both the Gα_q/11_ and β-arrestin pathways in a time-dependent manner to regulate Erk and localize pErk to the nucleus for downstream gene expression (68). Knock-in of ETR type A or type B in Gα_q/11_-deficient mice showed differential craniofacial development is based on specific ETR Gα_q_ and Gα_q/11_ requirements (69). Clearly, the Gα_q/11_ knockout mouse has proven itself a reliable and useful tool in the study of Gα protein-mediated signaling.

An alternative approach to exploring Gα protein signals has been to co-express Gα protein mutants or chimeras in different cell types. Gα_q_ chimeric mutants containing Gα_i_ or Gα_o_ tails co-expressed in COS-7 cells with opioid receptors and stimulated with opioid agonist are insensitive to pertussis toxin catalyzed ADP-ribosylation demonstrating an inability of Gα_i_ or Gα_o_ tails to serve as pertussis toxin substrates (70). Gi-coupled opioid receptors increase Gα_q_ signals as demonstrated by the co-expression of constitutively active Gα mutants in COS-7 cells and requires activated phospholipase beta and Gβγ dimers (71).

A considerable number of studies have explored membrane localization of Gα proteins in different cellular contexts and described a diversity of requirements. N-terminal sequence-mutated Gα proteins expressed in HEK293 cells are unable to localize to the plasma membrane due to their inability to bind to Gβγ or attach myristate and palmitate (72). Mutated Gα_q_ and Gα_s_ proteins deficient in Gβγ-binding and co-expressed with different β(1–5) or γ2/3 subunits show that Gβγ and Gα proteins promote membrane localization of the other and requires palmitoylation (73). Defects in plasma membrane localization of Gα_s_ occur when four N-terminal basic residues are mutated to glutamine; however, mutation of nine basic residues in Gα_q_ is required. Gβγ co-expression partially rescues localization indicating that the characteristics of the N-terminal residues of Gα_s_ and Gα_q_ are critical in membrane localization of these proteins (74). Using co-expressed constitutively active Gα_q_ or Gα_q/12_, the activation of ETRs was shown to mediate the binding of Gα_q_ or Gα_q/12_ in caveolae to enable the downstream activation of Erk1/2 (75).

Gα protein-mediated signaling studies across a wide variety of GPCRs dominate the literature using Gα protein mutants or chimeras. Constitutively active Gα_q_, Gα_q12_, or Gα_q13_ mutants transfected into Jurkat cells co-expressing GPCR muscarinic cholinergic receptor subtypes demonstrated a requirement for Gα_q13_ to activate downstream transcription factor serum response factor. However, the M1 subtype also required Gα_q/11_ and calcium when regulator proteins RGS2 and RGS4 were co-transfected that demonstrates a unique pathway for the M1 receptor (76). Gα_q/11_ (Y356D) mutation results in altered GPCR α1B-adrenoreceptor contact domain and abolishes receptor function, however, does not affect ligand binding (67). Studies using constitutively active mutants Gα_q_ (Q209L) and Gα_q/13_ (Q226L) demonstrate that Gα_q_ activates rat brain phospholipase D1; however, Gα_q/13_ inhibits its activity (77). Gα_q_ deletion mutants were used to demonstrate that Gα_q_ mediates down-regulation of the vesicle-associated GPCR vesicular monoamine transmitter transporter VMAT2 activity in platelets (78). Expression of a constitutively active Gα_q_ (R183C) mutant inhibited the expression of ezrin–radixin–moesin-binding phosphoprotein 50 and subsequent internalization of GPCR thromboxane A(2) beta receptor independently of PLC and PKC pathways (79). Specific Gα peptides and dominant negative Gα mutants were used to demonstrate the ability of α-thrombin to activate different effectors via Gα, Gβγ, and Gα_i2_, respectively, in Chinese hamster embryonic fibroblasts and thereby regulate the activation of the PI3kinase/Akt pathway (80). Molecular modeling and testing GST–fusion proteins of Gα_q_ mutants–GPCR kinase complexes revealed a critical residue Gαq Pro185 at the interface with GPCR kinase 2 with residues Gαq K77, L78, Q81, and R92 also playing key interactive roles (81). Constitutively active Gα_q_, Gα_q/12_, and Gα_q/13_ overexpressed in human astrocytoma cells increased agonist-activated thromboxane A2 receptor-mediated IL-6 production while mutated Gα_q_ and Gα_q/13_ overexpression blocks IL-6 production (82). Using both constitutively active and dominant negative Gα_q_ subunit expression showed that in neuroblastoma cells Gα_q_ elicits a rapid signal at the plasma membrane (83). Expression of constitutively active Gα_q_ (Q209L) mutant inhibits Ras and the PI3K/Akt pathway; however, minimal effects are seen on the Ras/Raf/MEK/Erk signaling pathway (84). Gα_q_ mutants that cannot bind Gβγ are unable to be stimulated by the mitogenic Pasteurellosis multocida toxin (PMT) demonstrating the requirement of cohesive Gα_q_/Gβγ signaling for this toxin activation pathway (85). Expressing GTPase-deficient Gα_q_ mutant in the human adrenal cell line H295R depolarizes the two-pore loop potassium channel TASK and thereby increases aldosterone secretion (86). Chimeric G proteins have been used to determine Gα responses from orphan GPCRs with unknown Gα coupling partners. A luciferase reporter system with a chimera that contains promoter elements that drive Gs, Gq, and G12 signals and another chimera with promoters to drive Gi signals revealed neuromedin U receptor 1 activating Gq, neuromedin U receptor 2 activating Gi, and luteinizing hormone receptor activating Gq and Gs proteins (87).

## Potential of Gα_q_ as a Therapeutic Target

Gα_q_ as a protein has several functions, which are valuable therapeutically. The GTPase activity, which hydrolyzes bound GTP to GDP, is an enzyme action that can be targeted. The binding of GDP and GTP are potential targets in the same manner in which the ATP binding site is target of many drugs inhibiting kinases (88). The ligand-activated GPCR acts as a GEF, which stimulates the exchange of GDP for GTP on the Gα peptide and this could be targeted. Furthermore, the protein contains a switch mechanism and this can be targeted as it is the target of the YM class of inhibitors (39). So, it is both theoretically possible and has been demonstrated that Gα_q_ can be exploited as a drug target.

The consequences of targeting signaling molecules have theoretical limitations based on the role of such targets in normal physiology but also conceptually there may be situations, pathophysiology, in which the activity of Gα_q_ is elevated or enhanced and presents itself as a target. Such situations are common in therapeutics but in most cases can only be established experimentally.

As discussed above, inhibition of Gα_q/11_ using YM-254890 has demonstrated anti-platelet aggregation, antithrombotic, and thrombolytic properties in a rat model of carotid artery thrombosis (40). Therefore, compounds that inhibit Gα_q/11_ could show enormous potential in the treatment of thrombotic conditions such as thrombotic stroke and myocardial infarction in humans. Additionally, a number of recent studies have also implicated a role for Gα_q/11_ in a range of metabolic conditions such as obesity and type 2 diabetes (89, 90). Activation of Gα_q_ results in pronounced increases in blood glucose levels in a mouse model (89), thus, compounds that inhibit Gα_q_ could also show promise as a future treatment option for type 2 diabetes.

In the important cardiovascular context of hypertension, Gα_q_ knockout mice have reduced blood pressure (41) and YM-254890 has demonstrated some anti-hypertensive properties (40). Although there are many effective anti-hypertensive agents currently available, there are also many subjects with medication-resistant hypertension, which does require a niche for new therapies although it is unclear if a Gα_q/11_ inhibitor would be suitable to consider for such a niche.

## Conclusion

G protein coupled receptor signaling is a major area of cell biology and therapeutics. The functioning of the seven transmembrane GPCR has been one of the most intensively studied areas of protein function. GPCRs signal through G proteins of the α and βγ subtypes where most of the signaling specificity is determined by the Gα protein. For the Gα protein family, these signaling pathways include the well-known PLC, PKC, and IP3 pathways and the lesser appreciated Rho/ROCK pathway. For multiple reasons, mostly the limited availability of pharmacological agents, which inhibit G protein function, the role of G proteins in GPCR signaling has been severely under-studied relative to the intense activity around the GPCRs. This is true for the Gαq proteins, which are the subject of this review but also for other G proteins. Given the broad involvement of GPCRs in cellular functioning, this is a major deficit in cellular signaling studies and potentially more importantly in the search for new drug targets. The recently expanding area of GPCR signaling is that of transactivation-dependent signaling in which GPCR transactivation of protein tyrosine and protein serine/threonine kinase cell surface receptors enormously expands the range of activities associated with the respective GPCRs. The potential role of G proteins and Gα proteins in particular in GPCR transactivation signaling is one very interesting area to be explored. It is likely that there are programs of chemical synthesis underway to synthesize inhibitors of Gαq proteins and these will increase the availability of inhibitors and also with the importance of this area hopefully lead to new studies, which produce a range of agents, some of which may be useful in *in vivo* studies. It is hoped that such studies may provide insights into the potential role of Gα_q_ in disease processes and reveal the extent to which such inhibitors may represent novel therapeutic agents in a range of conditions from cancer to cardiovascular disease.

## Author Contributions

PL, NO, VC, and WZ conceived the focus of the review, wrote, and edited the paper. HK and MB provided chemical insight about cyclic depsipeptide. DK, LT, and RB contributed expertise with preparation of the manuscript and the figures.

## Conflict of Interest Statement

The authors declare that the research was conducted in the absence of any commercial or financial relationships that could be construed as a potential conflict of interest.
